# Bioactive secondary metabolites from new endophytic fungus *Curvularia*. sp isolated from *Rauwolfia macrophylla*

**DOI:** 10.1371/journal.pone.0217627

**Published:** 2019-06-27

**Authors:** Fatma Kaaniche, Abdelaaty Hamed, Ahmed S. Abdel-Razek, Daniel Wibberg, Negera Abdissa, Imene Zendah El Euch, Noureddine Allouche, Lotfi Mellouli, Mohamed Shaaban, Nobert Sewald

**Affiliations:** 1 Department of Chemistry, Organic and Bioorganic Chemistry, Bielefeld University, Bielefeld, Germany; 2 Laboratory of Organic Chemistry, Natural Substances Team, Faculty of Sciences of Sfax, University of Sfax, Sfax, Tunisia; 3 Laboratory of Microorganisms and Biomolecules of the Centre of Biotechnology of Sfax-Tunisia, Sfax, Tunisia; 4 Chemistry Department, Faculty of Science, Al-Azhar University, Nasr City-Cairo, Egypt; 5 Microbial Chemistry Department, Genetic Engineering and Biotechnology Research Division, National Research Centre, Dokki-Giza, Egypt; 6 Center of Biotechnology(CeBiTec), Bielefeld University, Bielefeld, Germany; 7 Department of Chemistry, Jimma University, Jimma, Ethiopia; 8 Chemistry of Natural Compounds Department, Pharmaceutical and Drug Industries Research Division, National Research Centre, Dokki-Cairo, Egypt; Tallinn University of Technology, ESTONIA

## Abstract

Over the last decades, endophytic fungi represent a new source of pharmacologically active secondary metabolites based on the underlying assumption that they live symbiotically within their plant host. In the present study, a new endophytic fungus was isolated from *Rauwolfia macrophylla*, a medicinal plant from Cameroon. The fungus showed a highest homology to *Curvularia* sp. based on complete nucleotide sequence data generated from the internal transcribed spacer (ITS) of ribosomal DNA region. Large scale fermentation, working-up and separation of the strain extract using different chromatographic techniques afforded three bioactive compounds: 2'-deoxyribolactone (**1**), hexylitaconic acid (**2**) and ergosterol (**3**). The chemical structures of compounds **1–3** were confirmed by 1 and 2D NMR spectroscopy and mass spectrometry, and comparison with corresponding literature data. Biologically, the antimicrobial, antioxidant activities and the acetylcholinesterase inhibitory of the isolated compounds were studied.

## Introduction

*Rauwolfia macrophylla* (Apocynaceae) is a tree of about 12–15 m high occurring in Upper Guinea, Southern Nigeria, and Cameroon [1]. The stem and root barks were commonly used to treat malaria and other parasitic diseases in African indigenous medicine [2, 3]. Endophytes are microorganisms that inhabit the inner tissue of their hosts and perform various ecological relationships without showing visible host infection symptoms [4–6]. Isolation of three endophytic fungi namely *Aspergillus* sp., *Fusarium* sp., and *Purpureocillium lilacinum* from the stem bark of *Rauwolfia macrophylla* were recently reported [7]. The last endophytic fungus (*Purpureocillium lilacinum*) was among them specifically studied due to its potent antiprotozoal activity against *Leishmania donovani* (IC_50_ 0.174 μg mL^-1^); which was attributed to its production of purpureone, an ergochrome moiety [7]. It should be considered that different factors such as the plant organs, genotypic and geographic location influence the endophytic fungal community structure [8]. Endophytic fungi *Curvularia* sp. including *Curvularia trifolii* [9] and *Curvularia lunata* [10] provide a broad variety of bioactive secondary metabolites including polyketides and steroids. The genus *Curvularia* includs more than 40 species of saprophytes, endophytes and pathogens [11]. Most of these species are plant pathogens and give rise to losses in agricultural production [12].

In a continuing search for new endophytic fungi from medicinal plants as source of bioactive secondary metabolites, we isolated for the first time an endophytic fungus belonging to *Curvularia* sp. which has been characterized as *Curvularia* sp. T12 based on ITS sequencing (99% sequence identity with *Curvularia sorghina* BRIP 15900) from the stem bark of *Rauwolfia macrophylla* (Apocynaceae). An investigation of the fungus metabolites after its fermentation on solid-rice medium, working up and purification afforded three diverse classes of bioactive compounds. Identification of their structures on the bases of NMR and mass spectrometry assigned them as 2'-deoxyribolactone (**1**), hexylitaconic acid, (**2**) and ergosterol (**3**). The antibacterial, antioxidant, and acetylcholinesterase inhibitory activities of the compounds **1–3** were intensively reported as well.

## Materials and methods

### General

NMR spectra (^1^H NMR, ^13^C NMR, DEPT, COSY, HMQC, and HMBC) were measured on Bruker Avance DRX 500 spectrometer using standard pulse sequences and referenced to residual solvent signals. Column chromatography was carried out on silica gel 60 (0.040–0.063 mm, Merck, Darmstadt, Germany) and Sephadex LH-20 (GE Healthcare, Uppsala, Sweden) as stationary phases. ESI-MS was done on a Micromass AC-TOF micro mass spectrometer (Micromass, Agilent Technologies 1200 series, Tokyo, Japan). Analytical TLC was performed with pre-coated Merck silica gel 60 _PF254+366_ (Merck, Darmstadt, Germany). *R*_f_ values of the strain extract bioactive compounds and visualization of their chromatograms was carried out with UV light (254 and 366 nm) followed by spraying with anisaldehyde-sulfuric acid and heating. Acetylcholine iodide, AChE from electric eel (type VI-S lyophilized powder), 2,2-diphenyl-1-picrylhydrazyl (DPPH), 2,2’ azino-bis-(3-ethylbenzthiazoline-6-sulfonic acid (ABTS^+^), 5,5-dithiobis-2-nitrobenzoic acid (DTNB), BHA (butylated hydroxyanisole) and BHT (butylated hydroxytoluene), galanthamine were obtained from Sigma-Aldrich (Germany).

### Strain material

The fungal strain T12 was isolated from the stem bark of *Rauwolfia macrophylla* (Apocynaceae) collected from Mount Kalla in Cameroon. The stem surface of the plant was sterilized using the methods described by Pimentel et al. (2006) [13]. The stems were cleaned with distilled and sterilised water for 10 min to remove impurities and surface debris. After air-drying, the cleaned stems were cut into small pieces and then sterilized under aseptic conditions using 70% ethanol for 30 s, 2.4% sodium hypochlorite solution for 4 min, and then 70% ethanol for 30 s. The plant samples were finally washed (3 ×) with sterile distilled water for 1 min. The surface-sterilized samples were then further cut into smaller pieces (1 cm^2^) and aseptically placed in petri dishes containing sterile potato dextrose agar (PDA medium), supplemented with chloramphenicol (250 mg L^−1^) to inhibit bacterial growth. All plates were incubated at room temperature until mycelium grew out hyphal tips that were cut and transferred to PDA. The isolated strain was stored on PDA agar slants at 4 °C.

To identify the isolated endophytic fungal strain, the ITS region of the ribosomal DNA was analyzed by applying Sanger sequencing [14]. For DNA isolation, fungal cultures were grown in liquid potato dextrose broth (PDB) (24 g L^−1^, pH 7) for 5–7 days at room temperature. After centrifugation (12 000 ×*g*, 10 min, at room temperature), the mycelium pellet was shock frozen and ground with a pestle in liquid nitrogen. The fungal DNA was isolated using the DNeasy Plant Mini kit (Qiagen, Hilden, Germany). PCR mixtures to amplify the ITS regions contained 2.5 μL of 10 × PCR buffer (Promega, Fitchburg, MA, USA), 0.5 μL of dNTP mixture (10 mmol⋅L^−1^ each, NEB, Ipswich, MA, USA), 0.5 μL of each primer (10 μmol L^−1^, ITS1 (5′-TCC GTA GGT GAA CCT GCG G-3′) and ITS4 (5′-TCC TCC GCT TAT TGA TAT GC-3′) (White et al., 1990) [15], 0.5 μL of *Pfu* DNA polymerase (3 U μL−1, Promega), and 2 μL of isolated DNA in a total volume of 25 μL [15]. PCRs were carried out and the resulting products were purified using the QIAquick PCR purification kit (Qiagen) and sequenced on an Applied Biosystems Abi-Prism 3730 sequencer using BigDye-terminator chemistry at the Center for Biotechnology (CeBiTec, Bielefeld, Germany). For taxonomic classification, the resulting sequence was compared with the NT database (NCBI) by means of blastn and default settings. Sequences of the best hits were used for the construction of a phylogenetic tree. Phylogenetic analyses were conducted using MEGA version 7.0.2 [15, 16]. The phylogenetic tree was constructed by means of neighbourjoining (NJ) algorithm [17] using Kimura 20-parameter distance. The robustness of the inferred tree was evaluated by bootstrap (1000 replications).

### Fermentation, extraction and isolation

Inocula of the endophytic fungus *Curvularia* sp. T12 were introduced to five 1L-flasks, each containing sterilized solid-rice medium (100 g rice, 100 mL distilled water, followed by sterilization in an autoclave). The flasks were incubated at room temperature (25 °C) for 21 days. After fermentation, the obtained cultures were soaked in methanol (3 × 0.5 L per each flask for 24 h), followed by filtration. The filtered methanol extract was concentrated under vacuum at 40 °C to afford 60 g of brown crude extract.

**Fig 1:**
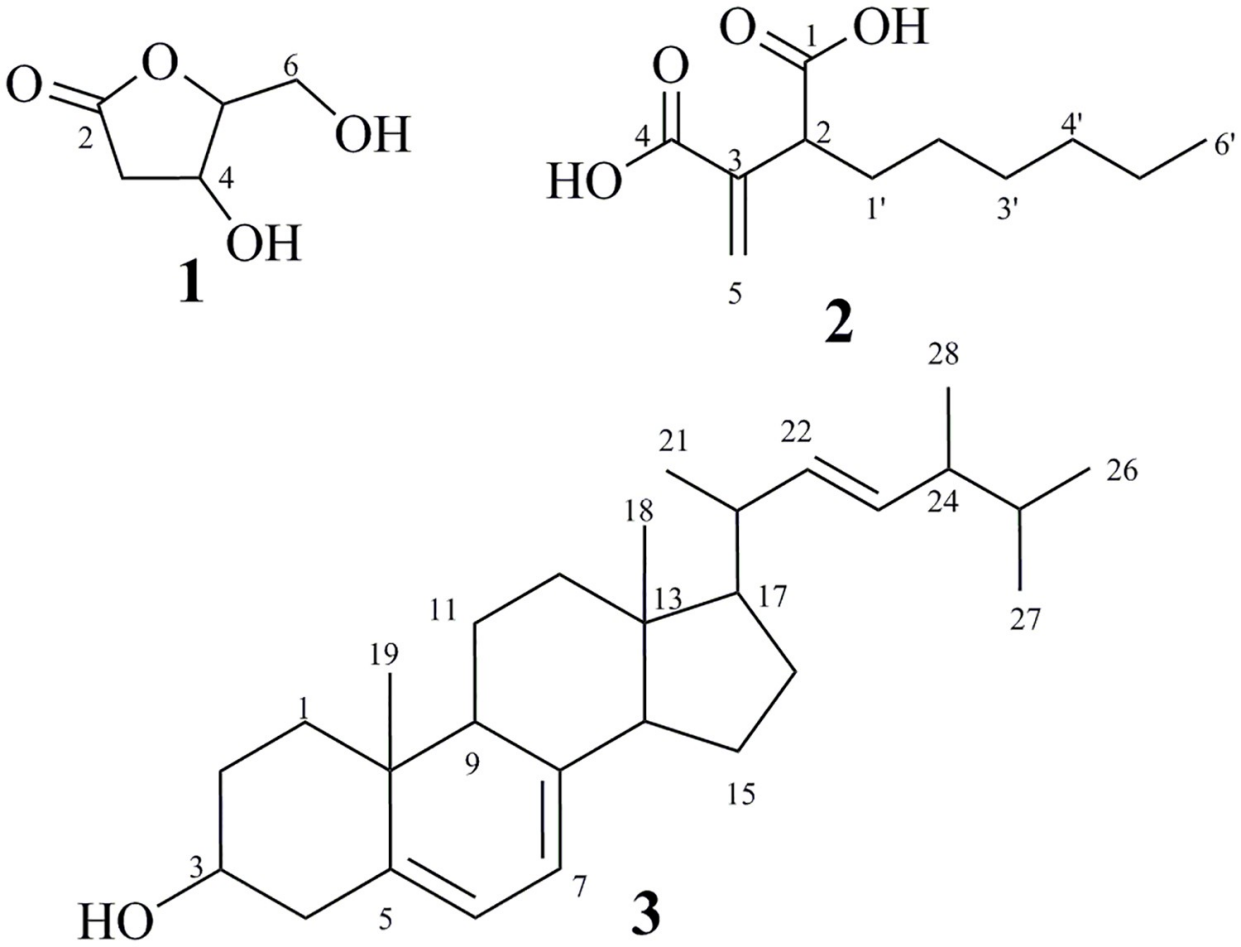
Structure of the compounds (1–3).

The extract was then applied to fractionation by silica gel column chromatography (80 × 10 cm) and eluted with petroleum ether-EtOAc-MeOH gradient with gradually increasing the polarity, affording six major fractions according to TLC monitoring: FI (2.9 g), FII (2.1 g), FIII (6.0 g), FIV (3.1 g), FV (5.0 g), FVI (8.1 g). Application of FI to silica gel column (60×1.5 cm) using cyclohexane-DCM gradient afforded ergosterol (**3**, 10 mg) as colorless solid. Further purification of fraction III was done on silica gel column chromatography eluted with petroleum ether/EtOAc (1:1) and followed by gel filtration using Sephadex LH-20 (DCM/MeOH, 1:1) resulting in colourless solids of 2'-deoxyribolactone (**1**, 1.3 mg) and hexylitaconic acid (**2**, 2.2 mg) (Fig 1).

**2'-Deoxyribolactone (1)**: colourless solid, UV-non-absorbing, showed yellow-green colour on spraying with anisaldehyde/sulfuric acid; *R*_f_ = 0.52 (DCM/5% MeOH); ^1^H NMR (500 MHz, CD_3_OD) and ^13^C NMR (125 MHz, CD_3_OD) (S1 Table, S1–S6 Figs); (+)-ESI-MS m/z = 155 ([M+Na]^+^); (-)-ESI-MS m/z = 131 ([M-H]^-^).

**Hexylitaconic acid (2)**: colourless solid, UV-absorbing (254 nm), showing brown colouration on spraying with anisaldehyde/sulphuric acid and heating; *R*_f_ = 0.39 (5% MeOH in DCM); ^1^H NMR (500 MHz, CDCl_3_) and ^13^C NMR (125 MHz, CDCl_3_) (S2 Table, S7–S12 Figs); (+)-ESI-MS m/z (%) = 237 ([M+Na]^+^); (-)-ESI-MS m/z (%) = 213 ([M-H]^-^).

**Ergosterol (3)**: Colourless amorphous solid, UV-absorbing (254 nm), purple to dark blue colour on spraying with anisaldehyde/sulfuric acid; *R*_f_ = 0.48 (3% MeOH in DCM); ^1^H NMR (500 MHz, CDCl_3_, S13 Fig) *δ* = 5.50 (dd, *J* = 5.7, 2.5 Hz, 1H), 5.31 (dt, *J* = 5.6, 2.8 Hz, 1H), 5.16 (dd, *J* = 15.2, 7.0 Hz, 1H), 5.10 (dd, *J* = 15.3, 7.7 Hz, 1H), 3.57 (tt, *J* = 11.2, 4.0 Hz, 1H), 2.40 (ddd, *J* = 14.3, 4.8, 2.4 Hz, 1H), 2.22 (ddt, *J* = 14.2, 11.8, 2.3 Hz, 1H), 2.03–1.93 (m, 2H), 1.91 (dddd, *J* = 11.9, 7.2, 4.6, 2.2 Hz, 1H), 1.80 (tdd, J = 13.9, 12.8, 6.4, 4.6 Hz, 4H), 1.73–1.48 (m, 4H), 1.48–1.36 (m, 2H), 1.36–1.13 (m, 6H), 0.97 (d, *J* = 6.6 Hz, 3H), 0.88 (s, 3H), 0.85 (d, *J* = 6.9 Hz, 3H), 0.76 (t, *J* = 7.2 Hz, 6H), 0.56 (s, 3H). ^13^C NMR (125 MHz, CDCl_3_, S14 Fig) *δ* 141.3, 139.8, 135.6, 132.0, 119.6, 116.3, 70.4, 55.7, 54.6, 46.2, 42.8, 40.8, 40.4, 39.1, 38.4, 37.0, 33.1, 32.0, 28.3, 23.0, 21.1, 20.0, 19.6, 17.6, 16.3, 12.0. (+)-ESI-MS m/z (%) = 419 ([M+Na]^+^); (-)ESI-MS m/z (%) = 395 ([M-H]^-^).

### *In vitro* antibacterial assay

The antimicrobial activity of the isolated compounds was studied using micro-dilution method with some modification [18]. The bacterial strains, *Escherichia coli* (DSMZ1058), *Micrococcus luteus* (DSMZ1605), *Pseudomonas agarici* (DSMZ11810), and *Staphylococcus warneri* (DSMZ20036) were grown on a nutrient agar medium (3 g L^−1^ beef extract, 10 g L^−1^ peptone, and 20 g L^−1^ agar) and the pH was adjusted to 7.2. The test was performed by inoculating a suspension of the overnight tested microorganism (DO_600_ = 0.05 ~ 0.1) on the nutrient agar medium. The investigated compounds were diluted to 100 μL of broth in the first well of 96 wells of microtiter plate. The concentrations of the compounds were finally in the range of 250 mg mL^-1^ to 0.01 mg mL^-1^. Microtiter plates containing *Escherichia coli* and *Staphylococcus warneri* were incubated at 37°C and those containing *Micrococcus luteus* and *Pseudomonas agarici* were incubated at 30 °C for 24 h. The minimum inhibition concentration (MIC) was calculated according to our previous work [19].

### *In vitro* acetylcholinesterase inhibition assay

AChE inhibitory activity was measured according to the spectrophotometric method of Ellman with slight modifications [20]. Electric eel AChE was used, while acetylthiocholine iodide (ATCI) was employed as substrate. 5.5'-Dithiobis-(2-nitrobenzoic acid) (DTNB) was used for the determination of released thiocholine. Briefly, in this method, 100 μL of Tris buffer at 50 mM (pH 8.0), 30 μL of sample (100 μg/mL) and 5 μL of AChE enzyme (0.5 U/mL) were added in a 96 well microplate and incubated for 10 min at 25 °C. Then, 27 μL of DTNB (3 mM) and 23 μL of substrate (1.16 mM) were added. Hydrolysis of ATCI was monitored by the formation of the yellow 5-thio-2-nitrobenzoate anion as a result of the reaction of DTNB with thiocholines, at 405 nm utilizing a 96 well microplate reader (Thermo Scientific/Varioskan Flash, Germany). The reaction progress was monitored until the AChE activity decreased, and then the reaction has been stopped. Inhibition percentage was calculated according to Michaelis–Menten model by using *EZ-Fit*. The IC_50_ values were determined by monitoring the inhibition effects of various concentrations of under investigation compounds and calculated EZ-Fit, Enzyme Kinetics Program (Perrella Scientific In., Amherst, USA). Galanthamine dissolved in 10% DMSO in Tris buffer were used as standard drugs at 10 μg mL^-1^ and 1 mg mL^-1^ concentrations.

### *In vitro* antioxidant assays

#### DPPH and (ABTS^+^) free radical scavenging activities

The DPPH and ABTS^+^ assays [21, 22] were used to evaluate the free radical scavenging activities of the compounds. Briefly, the compounds were dissolved in 10% DMSO and diluted at different concentrations of 25 to 1 μgmL^-1^. For the DPPH assay, 500 μL of a 4% (w/v) solution of DPPH radical in methanol was mixed with 500 μL of samples under investigation. The mixture was incubated for 30 min in the dark at room temperature. The scavenging capacity was determined spectrophotometrically by monitoring the decrease in absorbance at 517 nm against a blank. For the ABTS^+^ assay, the reaction mixture was containing ABTS solution (7 mM) with 2.45 mM K_2_S_2_O_8_. The mixture was let to stand for 15 minutes in the dark at room temperature. The solution was diluted with ethanol to obtain the absorbance of 0.7 ± 0.2 units at 734 nm. In order to measure the antiradical activity of extracts and standards, 20 μL of each sample at various concentrations was added to 180 μL of ABTS^+^ and vortexed for 30 s, and then incubated for 6 min in the dark at room temperature. The absorbance was measured spectrophotometrically at 734 nm. The percentage of antioxidant activity had been calculated as follows:

$$Antioxidant\;activity\;\left(\%\right)=[(A_{control}-\;A_{sample})/A_{control}]\;x\;100.$$

BHA and BHT were used as positive controls.

### Data analyses

Curve-fitting analyses was made using GraphPad Prism version 4.03 (GraphPad Software, San Diego, CA, USA) [23].

## Results and discussion

### Taxonomical characterization of the producing fungus

**Fig 2:**
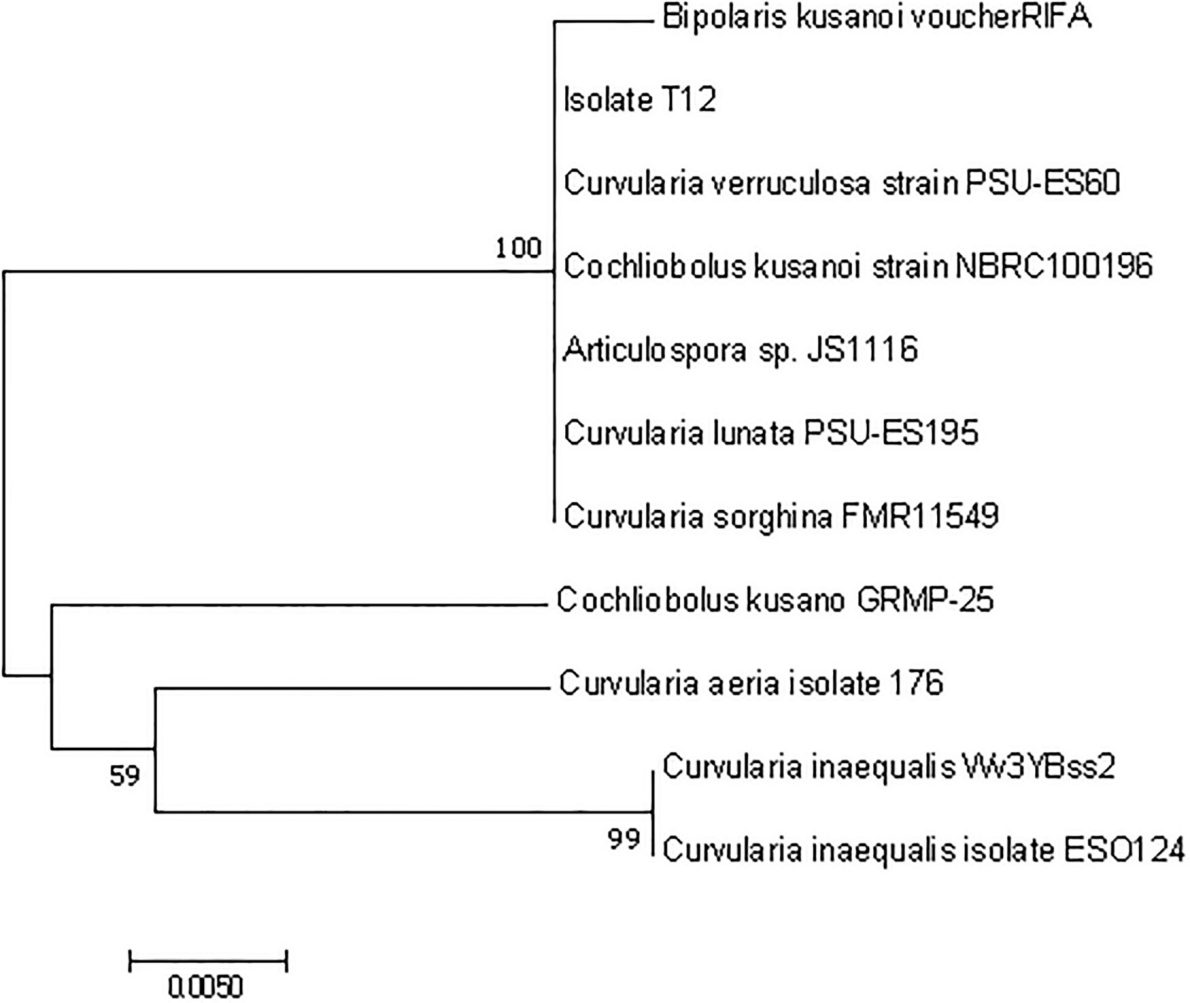
Phylogenetic tree of the endophyte strain T12.

An endophytic fungus was isolated from *Rauwolfia macrophylla* (Apocynaceae). For taxonomic classification of this endophyte, its ITS sequences were amplified by applying PCR, followed by Sanger sequencing and a BLAST-based approach. The obtained sequences showed hits against the databases with high identities (99%–100%) and sequence coverage (97%–100%). Best hits were selected to construct a phylogenetic tree (Fig 2). Based on the phylogenetic and the BLAST analyses, the fungal strain named T12 was assigned as *Curvularia* sp., since the closest available strains in the NCBI database are belonging to *Curvularia* spp, and the most similar one is *Curvularia sorghina* (99% sequence identity).

### Fermentation and structural identification

The extract produced by the endophytic fungus *Curvularia* sp. T12 was found to exhibit an antimicrobial inhibition zones (IZ) against *Escherichia coli* (IZ = 14.7 ± 1.0 mm), *Micrococcus luteus* (IZ = 14.0 ± 0.5 mm), *Pseudomonas agarici* (IZ = 12.3 ± 0.6 mm), and *Staphylococcus warneri* (IZ = 17.0 ± 1.5 mm), which represent a strong antimicrobial activities against these tested microbes. Accordingly, the fungus T12 was subjected to large scale fermentation to produce reasonable amounts of crude extract, After then, the crude extract has been applied for working up and purification of its desired metabolites using a series of chromatographic techniques, starting with silica gel column chromatography and ended by Sephadex LH-20. Based on that, three diverse bioactive compounds (**1–3**) were obtained.

The chemical structures of the isolated metabolites were determined as 2'-deoxyribolactone (**1**) [24–27], hexylitaconic acid (**2**) [28–30], ergosterol (**3**) [31–33] based on comparing their 1D & 2D nuclear magnetic resonance and ESI-MS data with those previously reported in literature (see experimental section and supplementary data).

#### Ribolactone derivatives

Synthetically developed and natural ribolactones derivatives are included as building blocks of several antiviral regimens [34]. The diversity and availability of these relatively cheap chiral compounds has led to their use as starting materials for the design and syntheses of naturally occurring compounds and biologically important molecules [35, 36].

#### Hexylitaconic acid derivatives

Itaconic acid derivatives (IA) represent promising compounds that have a wide range of applications and can be obtained in large scale using fermentation processes. One of the most important uses of these biomonomer is the environmentally sustainable production of biopolymers. IA is used worldwide in the industrial synthesis of resins such as polyesters, plastics and artificial glass and in the preparation of bioactive compounds in the agriculture, pharmacy and medicine sectors. IA also provides possibilities for selective enzymatic transformations to create useful polyfunctional building blocks [37]. IAs were found to possess significant anti-inflammatory and analgesic activities as well [38]. Moreover, hexylitaconic acid (2) has been reported as inhibitor of p53-HDM2 interaction [39].

#### Ergosterol and structural analogues

Ergosterol is the major product of sterol biosynthesis in fungi, and also in some trypanosomes. Most of the current antifungal agents interfere with ergosterol-function in some way, either through inhibition of various steps in ergosterol biosynthesis (allylamines, azoles, morpholines) or by complexing directly with membrane ergosterol (polyenes). Biologically, ergosterol was reported to have several effective activities, for example it has an inhibitory effect on the activity of mucosal-type mast cells [40], anti-*Trypanosoma cruzi* activity against trypomastigotes (IC_50_ 51.3 μg/mL) [41], and antifungal activity as well. More recently, ergosterol derivatives were reported as Anti-tumor and Anti-angiogenic agents [42].

### Biological activity studies

The isolated compounds **1**–**3** were evaluated for antimicrobial activity against four bacterial strains (*Escherichia coli*, *Micrococcus luteus*, *Pseudomonas agarici* and *Staphylococcus warneri*). The antibacterial activity test (Table 1) indicated that compounds **1** and **2** have antibacterial activities against all the tested bacterial strains with MIC ranging between 0.17 μg mL^-1^ and 0.58 μg ml^-1^.

**Table 1:** MIC (μg ml^-1^.) of the isolated compounds (1–3).

	*Pseudomonas agarici*	*Escherischia coli*	*Staphylococcus warneri*	*Micrococcus luteus*
**MIC (μg/ml)**				
**1**	0.19	0.30	0.17	0.32
**2**	0.29	0.58	0.58	0.29
**3**	-	-	-	-
**Gentamicin**^**a**^	0.01	0.01	0.01	0.01

MIC = Minimal inhibition concentration, Minimal, (*) = European Pharmacopoeia (EP) Reference Standard.

The isolated compounds (**1–3**) were evaluated also as inhibitors of acetylcholinesterase. They showed good inhibitory potential towards acetylcholinesterase (Table 2) with IC_50_ values of 1.93, 1.54 and 1.52 μM, respectively, while the selected reference galanthamine showed an IC_50_ value of 0.5 μM and 0.01 μM for tacrine.

**Table 2:** Acetylcholinesterase inhibition of compounds (1–3).

	1	2	3	Galanthamine*	Tacrine*
IC_50_ (μM)	1.93	1.54	1.52	0.5	0.01

**IC**_**50**_ = Concentration of acetylcholinesterase inhibitor drug that gives half maximal response, (*) = European Pharmacopoeia (EP) Reference Standard.

Antioxidant activity of the three compounds was measured according to DPPH and ABTS^+^ scavenging methods [43]. The results (Table 3) showed that compounds **1**–**3** were active with EC_50_ values 0.66, 0.56 and 1.09 μM, respectively in DPPH assay and 0.49, 0.88 and 0.83 μM in ABTS assay. Compounds **1** and **2** exhibited close EC_50_ values to those of the reference BHT (EC_50_ = 0.46 μM in DPPH assay), reflecting the high scavenging ability of the compounds.

**Table 3:** Antioxidant and radical scavenging capacity of the compounds (1–3).

	1	2	3	BHA*	BHT*
	**EC**_**50**_				
Antioxidant activity (**DPPH**^**•**^)	0.66 ±0.02	0.56 ±0.02	1.09 ±0.02	0.13	0.46
Antiradical activity (**ABTS**^**+**^)	0.49±0.02	0.88 ±0.02	0.83 ±0.02	0.01	0.06

**EC**_**50**_ = Concentration of an antioxidant drug that gives half maximal response, (*) = European Pharmacopoeia (EP) Reference Standard.

Numerous diverse bioactive compounds have been recently isolated from the endophyte *Curvularia* spp. indicating their importance as talented and major sources of bioactive molecules [9, 44]. However, the fungal metabolites have increased the interest in biomolecules activity studies [10].

## Conclusion

The new endophytic fungus *Curvularia* sp. T12 was isolated from the medicinal plant *Rauwolfia macrophylla*. Its Large scale fermentation and working up of the crude extract, led to the isolation of 2'-deoxyribolactone (**1**), hexylitaconic acid (**2**) and ergosterol (**3**). Compounds **1**–**3** displayed antimicrobial, antioxidant and acetylcholinesterase inhibition activities. To our knowledge, these biological activities have never been assayed for these compounds. These findings are greatly highlighting the importance of endophytic fungi as source of bioactive secondary metabolites.

## Supporting information

S1 Table ^13^C (125 MHz) and ^1^H (500 MHz) NMR data of 2'-deoxyribolactone (1) in CD_3_OD. (PDF)

S2 Table ^13^C (125 MHz) and ^1^H (500 MHz) NMR data of hexylitaconic acid (2) in CDCl_3_. (PDF)

S1 Fig ^1^H NMR spectrum (CD_3_OD, 500 MHz) of 2'-deoxyribolactone (1). (PDF)

S2 Fig ^13^C NMR spectrum (CD_3_OD, 125MHz) of 2'-deoxyribolactone (1). (PDF)

S3 Fig DEPT spectrum (CD_3_OD, 125MHz) of 2'-deoxyribolactone (1). (PDF)

S4 Fig H,H-COSY spectrum (CD_3_OD, 500 MHz) of 2'-deoxyribolactone (1). (PDF)

S5 Fig HMQC spectrum (CD_3_OD, 500 MHz) of 2'-deoxyribolactone (1). (PDF)

S6 Fig HMBC spectrum (CD_3_OD, 500 MHz) of 2'-deoxyribolactone (1). (PDF)

S7 Fig ^1^H NMR spectrum (CDCl_3_, 500 MHz) of Hexylitaconic acid (2). (PDF)

S8 Fig ^13^C NMR spectrum (CDCl_3_, 125MHz) of Hexylitaconic acid (2). (PDF)

S9 Fig DEPT spectrum (CDCl_3_, 125MHz) of Hexylitaconic acid (2). (PDF)

S10 Fig H,H-COSY spectrum (CDCl_3_, 500 MHz) of Hexylitaconic acid (2). (PDF)

S11 Fig HMQC spectrum (CDCl_3_, 500 MHz) of Hexylitaconic acid (2). (PDF)

S12 Fig HMBC spectrum (CDCl_3_, 500 MHz) of Hexylitaconic acid (2). (PDF)

S13 Fig ^1^H NMR spectrum (CDCl_3_, 500 MHz) of ergosterol (3). (PDF)

S14 Fig ^13^C NMR spectrum (CDCl_3_, 125MHz) of ergosterol (3). (PDF)

S1 File Raw data. (RAR)
