# Supplementary material for: Bioactive secondary metabolites from new endophytic fungus Curvularia. sp isolated from Rauwolfia macrophylla
Source: PLoS One. 2019 Jun 27;14(6):e0217627. doi: 10.1371/journal.pone.0217627 (PMC6597039; doi:10.1371/journal.pone.0217627)
Supplement: S1 Table — (PDF) [file pone.0217627.s001.pdf]

S1 Table. ^{13}C (125 MHz) and ^{1}H (500 MHz) NMR data of 2'-deoxyribolactone (**1**) in CD_3OD

**No.**	δ_C	δ_H (*J* in Hz)
2	177.4	-
3	37.7	2.92 (dd, 18.0, 6.8), 2.37 (dd,18.0, 2.5)
4	68.3	4.43 (dt, 6.7, 2.3)
5	88.8	4.37 (td, 3.4, 2.2)
6	61.1	3.76 (dd, 12.4, 3.3), 3.69 (dd, 12.5, 3.6)
