# Supplementary material for: Bioactive secondary metabolites from new endophytic fungus Curvularia. sp isolated from Rauwolfia macrophylla
Source: PLoS One. 2019 Jun 27;14(6):e0217627. doi: 10.1371/journal.pone.0217627 (PMC6597039; doi:10.1371/journal.pone.0217627)
Supplement: S2 Table — (PDF) [file pone.0217627.s002.pdf]

S2 Table. ^{13}C (125 MHz) and ^{1}H (500 MHz) NMR data of hexylitaconic acid (**2**) in $CDCl_3$

**No.**	δ_C	δ_H (*J* in Hz)
1	178.9	-
2	47.4	3.32 (t, 7.5)
3	137.6	-
4	171.1	-
5	129.1	6.41 (s), 5.74 (s)
1'	29.6	1.85 (m), 1.65 (d, 10.5)
2'	27.3	1.24 (m)
3'	29.0	1.24 (m)
4'	31.6	1.20 (m)
5'	22.6	1.20 (m)
6'	14.0	0.81 (t, 6.6)
