# Supplementary figures and images for: Bioactive secondary metabolites from new endophytic fungus Curvularia. sp isolated from Rauwolfia macrophylla
Source: PLoS One. 2019 Jun 27;14(6):e0217627. doi: 10.1371/journal.pone.0217627 (PMC6597039; doi:10.1371/journal.pone.0217627)

**S1 Fig.**  $^1\text{H}$  NMR spectrum ( $\text{CD}_3\text{OD}$ , 500 MHz) of 2'-deoxyribolactone (**1**)

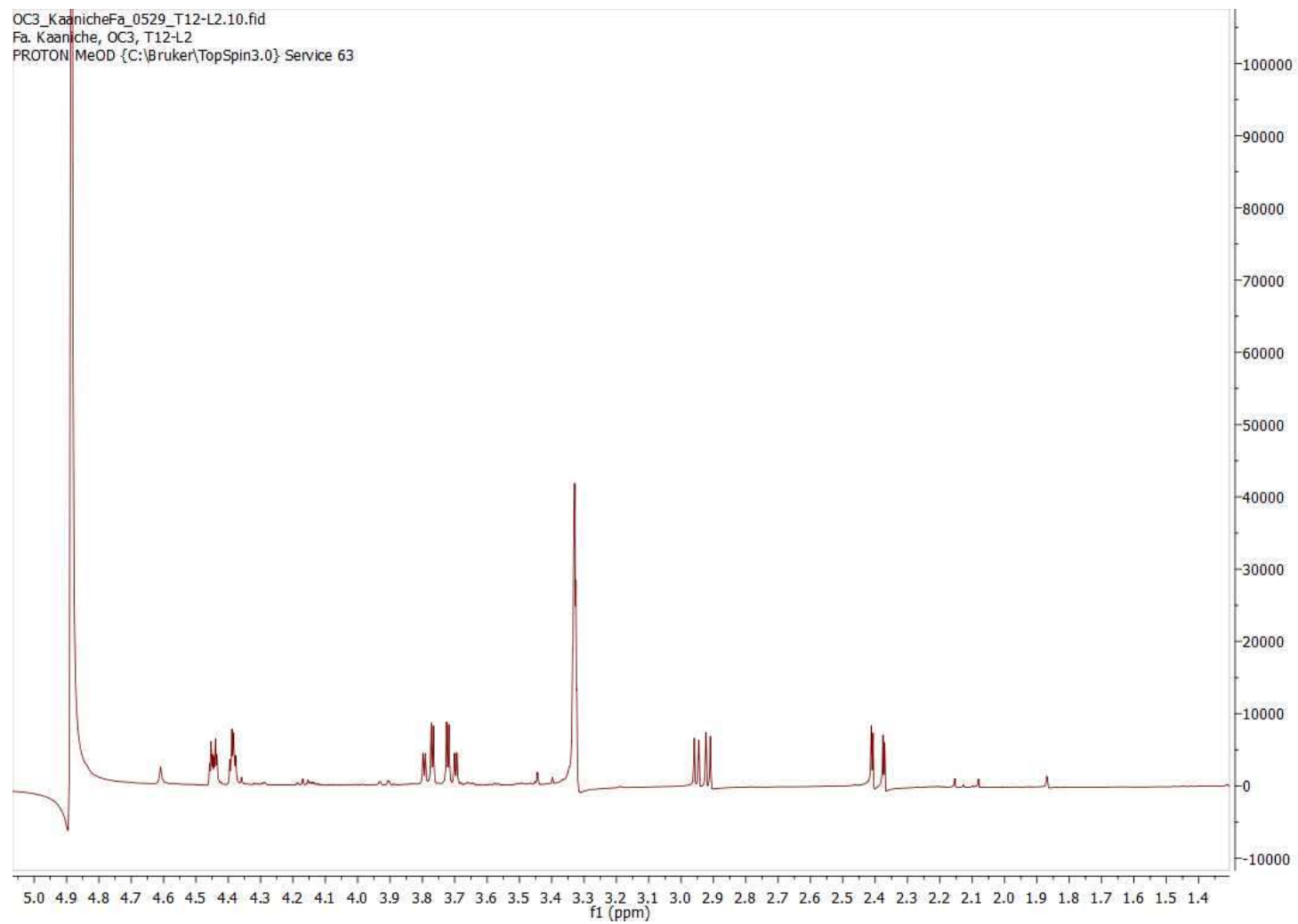

Supplement: S1 Fig — (PDF) [file pone.0217627.s003.pdf]

**S2 Fig.**  $^{13}\text{C}$  NMR spectrum ( $\text{CD}_3\text{OD}$ , 125MHz) of 2'-deoxyribolactone (**1**)

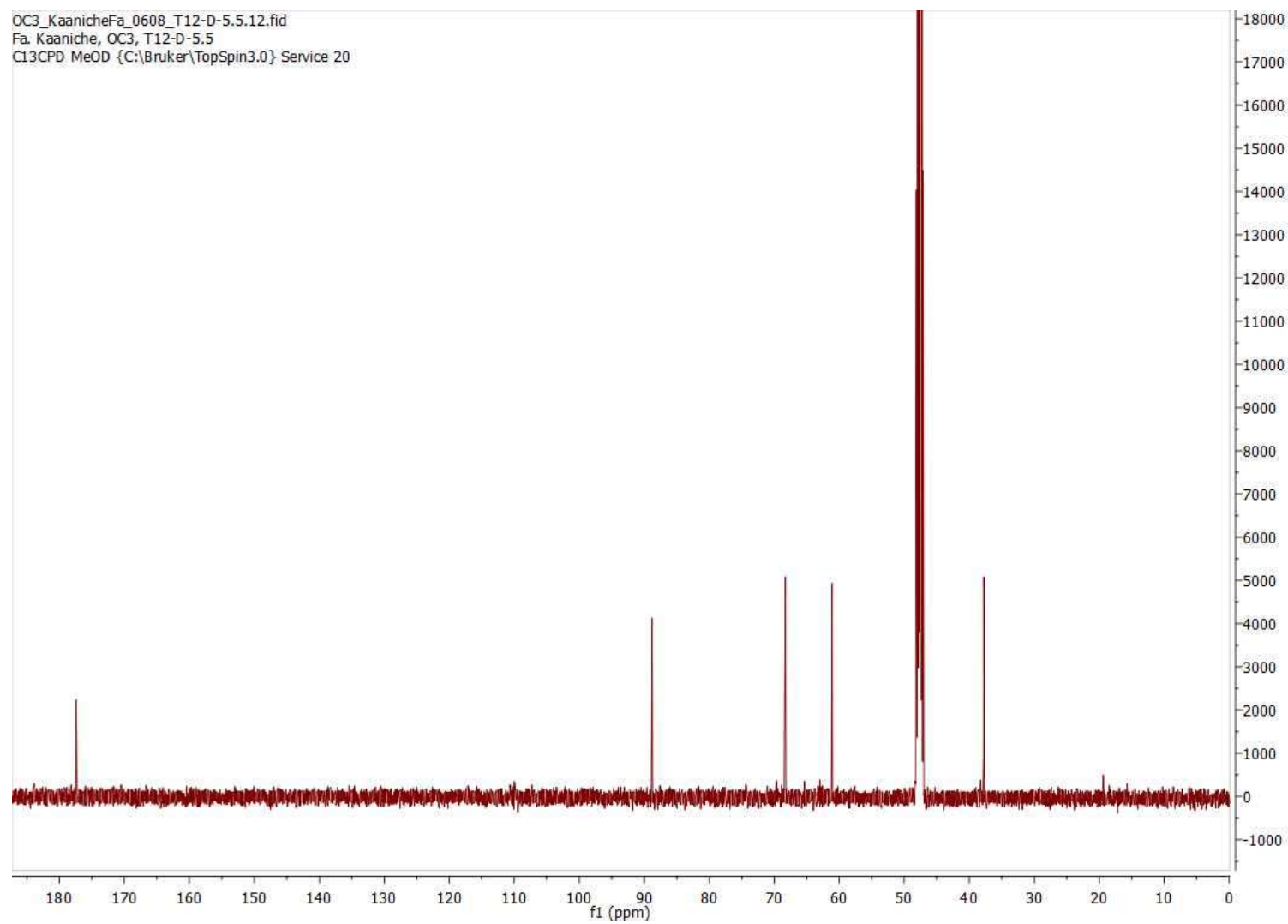

Supplement: S2 Fig — (PDF) [file pone.0217627.s004.pdf]

**S4 Fig.** H,H-COSY spectrum (CD<sub>3</sub>OD, 500 MHz) of 2'-deoxyribolactone (**1**)

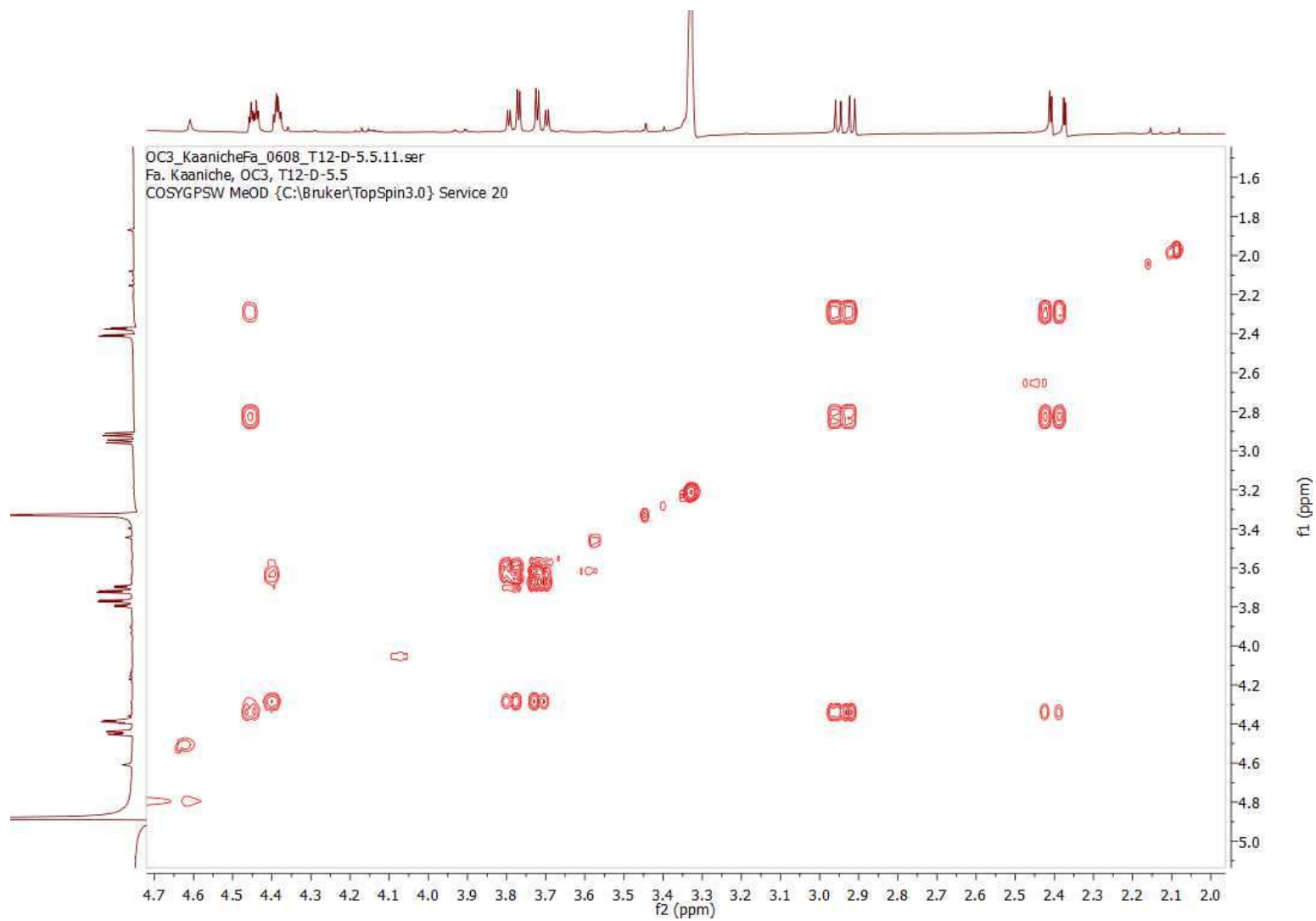

Supplement: S3 Fig — (PDF) [file pone.0217627.s005.pdf]

**S4 Fig.** H,H-COSY spectrum (CD<sub>3</sub>OD, 500 MHz) of 2'-deoxyribolactone (**1**)

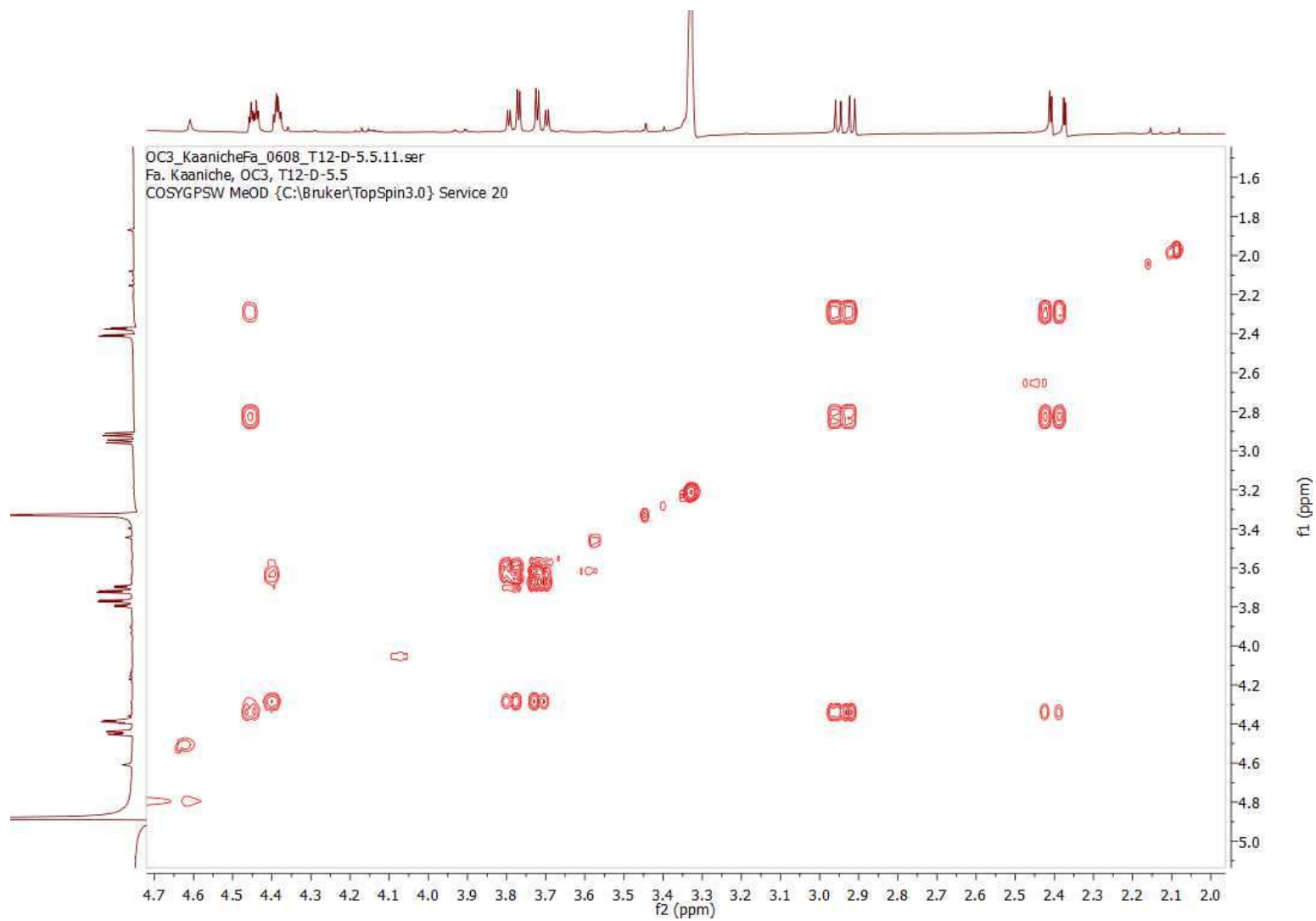

Supplement: S4 Fig — (PDF) [file pone.0217627.s006.pdf]

**S5 Fig.** HMQC spectrum (CD<sub>3</sub>OD, 500 MHz) of 2'-deoxyribolactone (**1**)

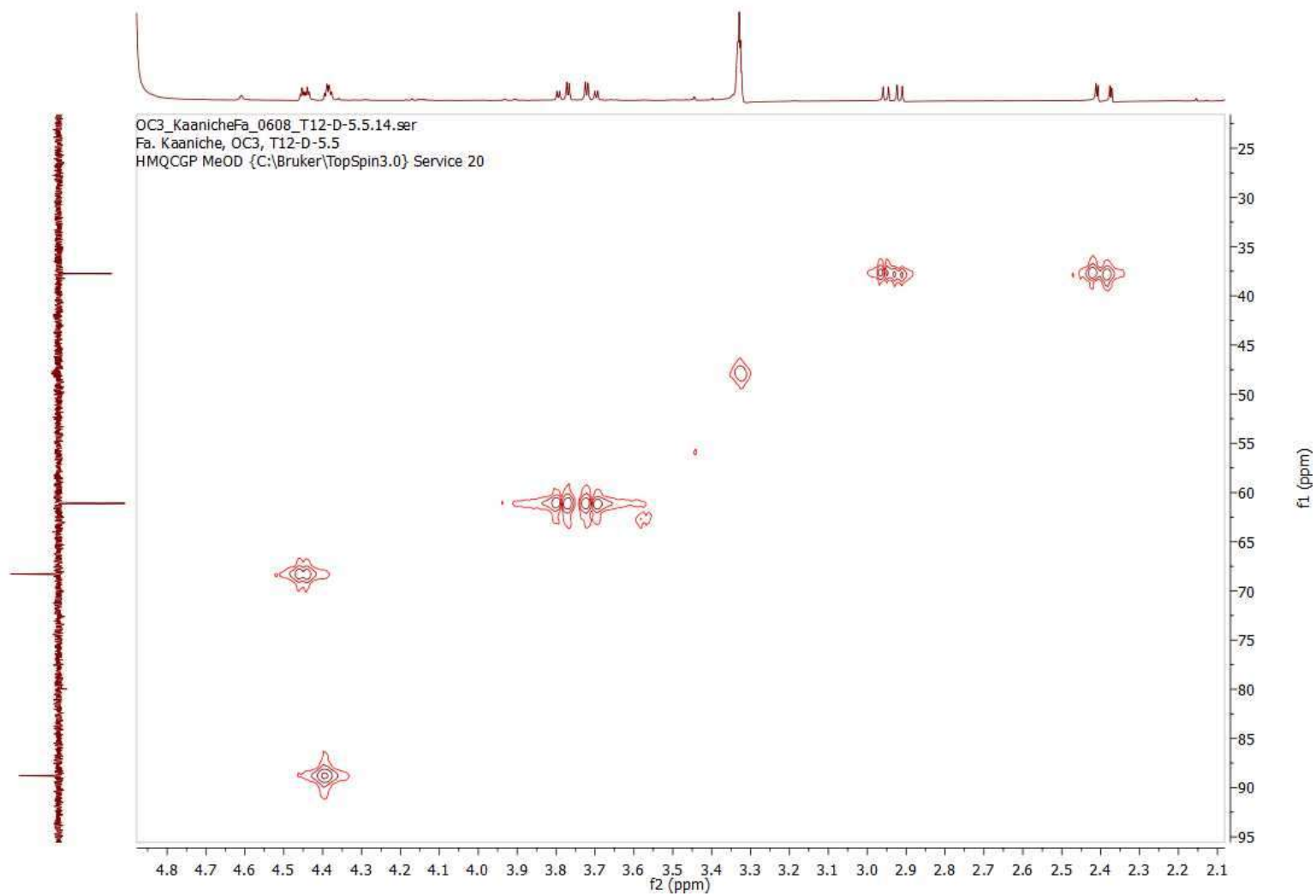

Supplement: S5 Fig — (PDF) [file pone.0217627.s007.pdf]

**S6 Fig.** HMBC spectrum (CD<sub>3</sub>OD, 500 MHz) of 2'-deoxyribolactone (**1**)

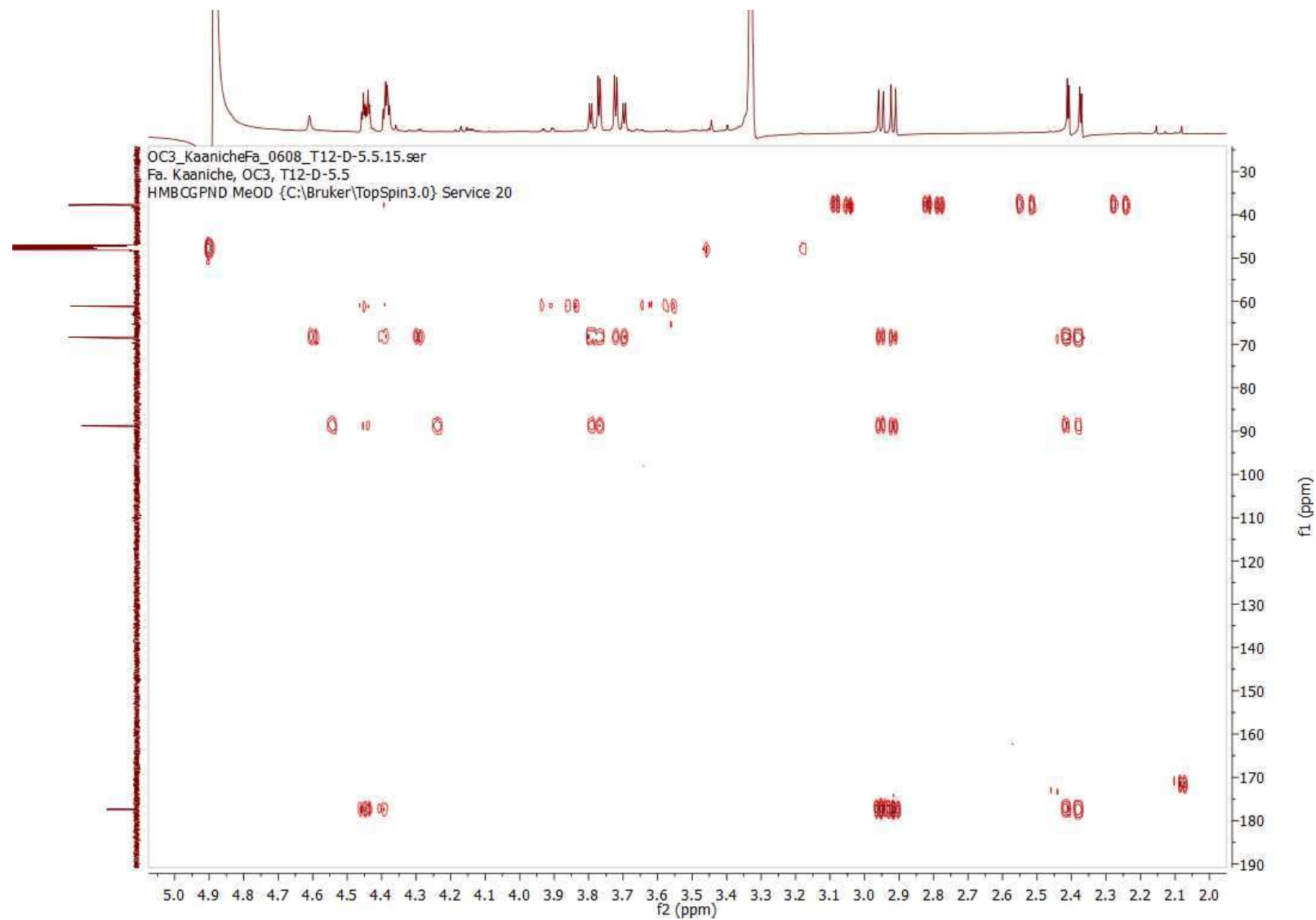

Supplement: S6 Fig — (PDF) [file pone.0217627.s008.pdf]

**S7 Fig.**  $^1\text{H}$  NMR spectrum ( $\text{CDCl}_3$ , 500 MHz) of Hexylitaconic acid (**2**)

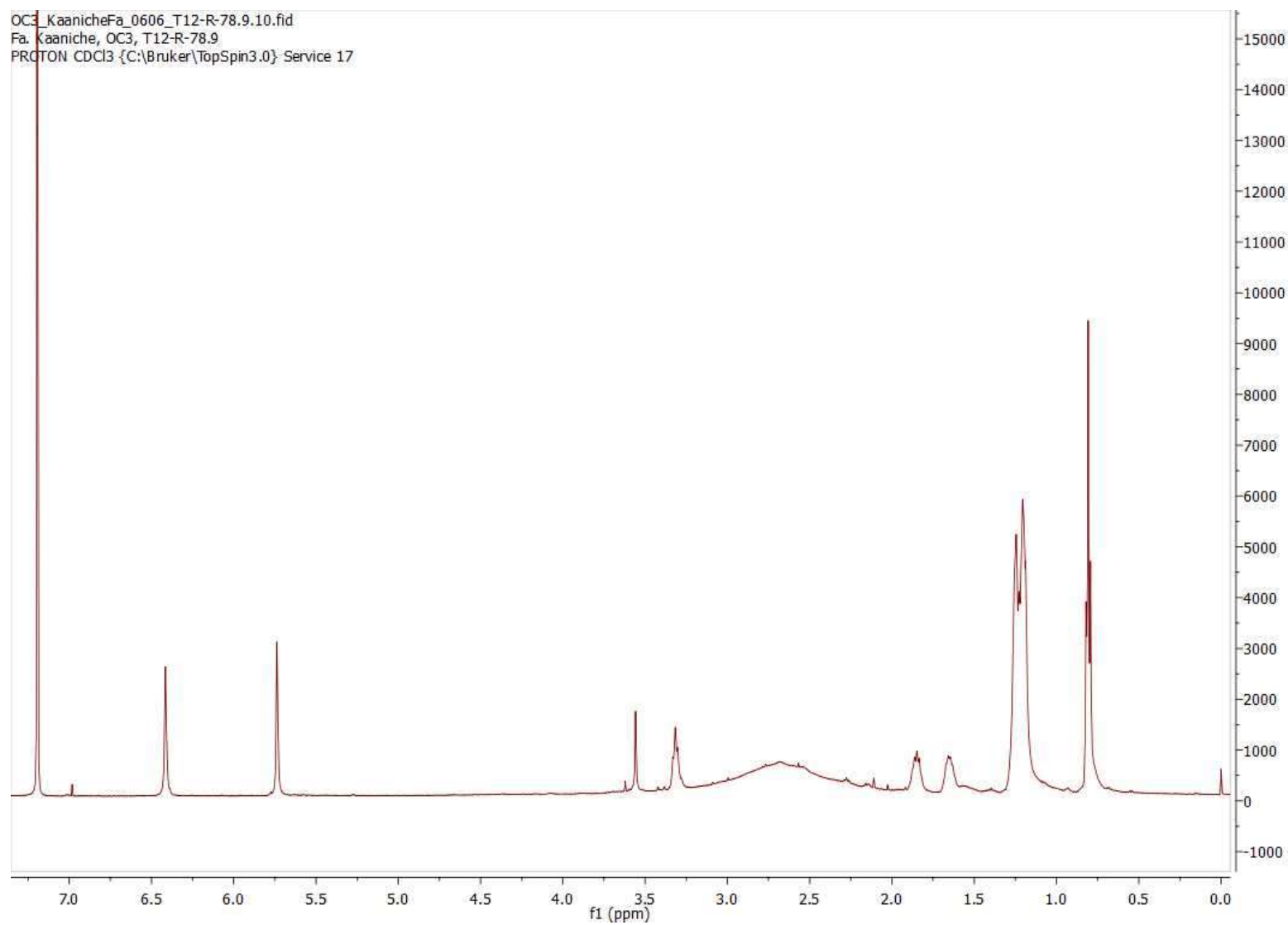

Supplement: S7 Fig — (PDF) [file pone.0217627.s009.pdf]

**S8 Fig.**  $^{13}\text{C}$  NMR spectrum ( $\text{CDCl}_3$ , 125MHz) of Hexylitaconic acid (**2**)

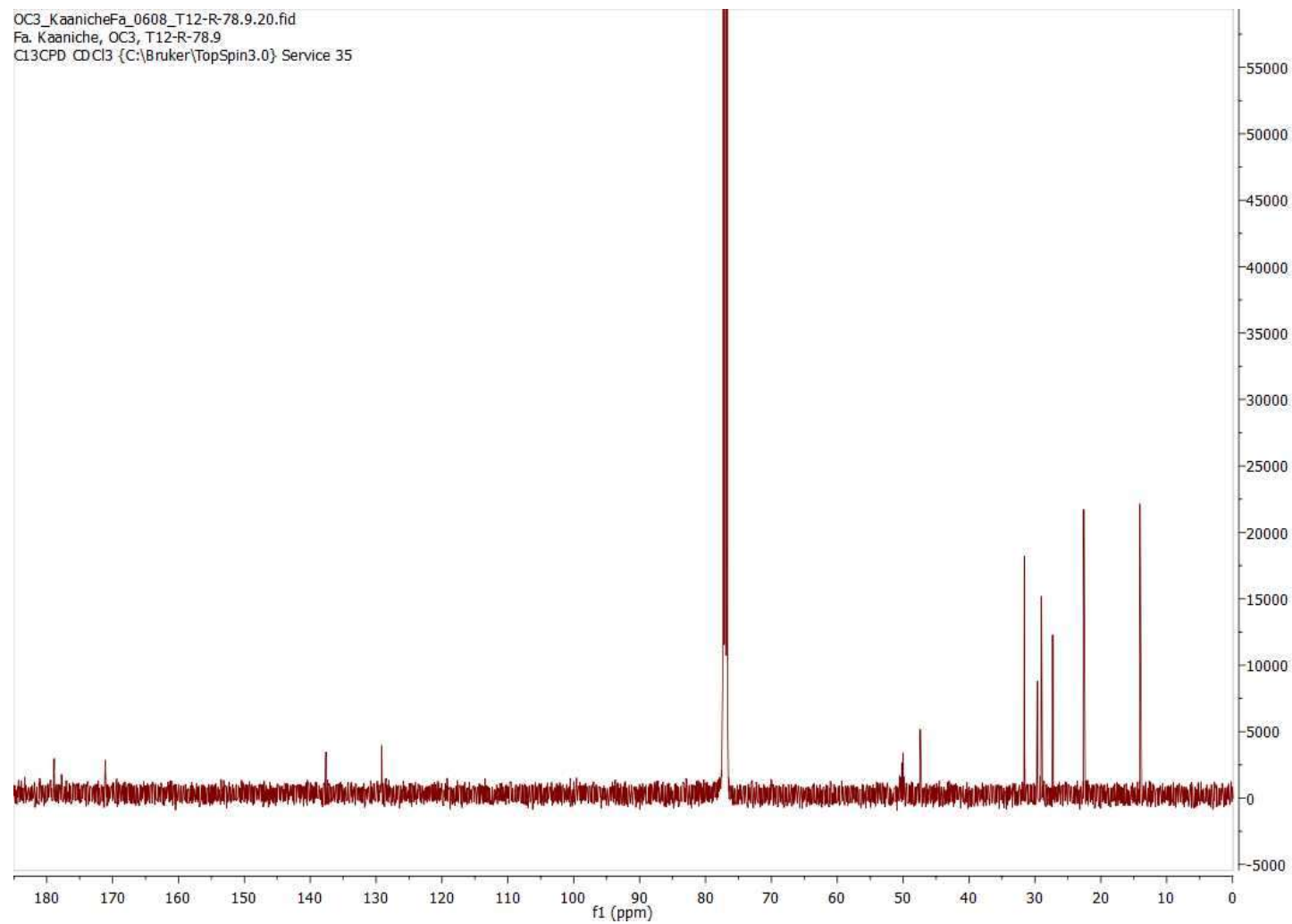

Supplement: S8 Fig — (PDF) [file pone.0217627.s010.pdf]

**S9 Fig.** DEPT spectrum (CDCl<sub>3</sub>, 125MHz) of Hexylitaconic acid (2)

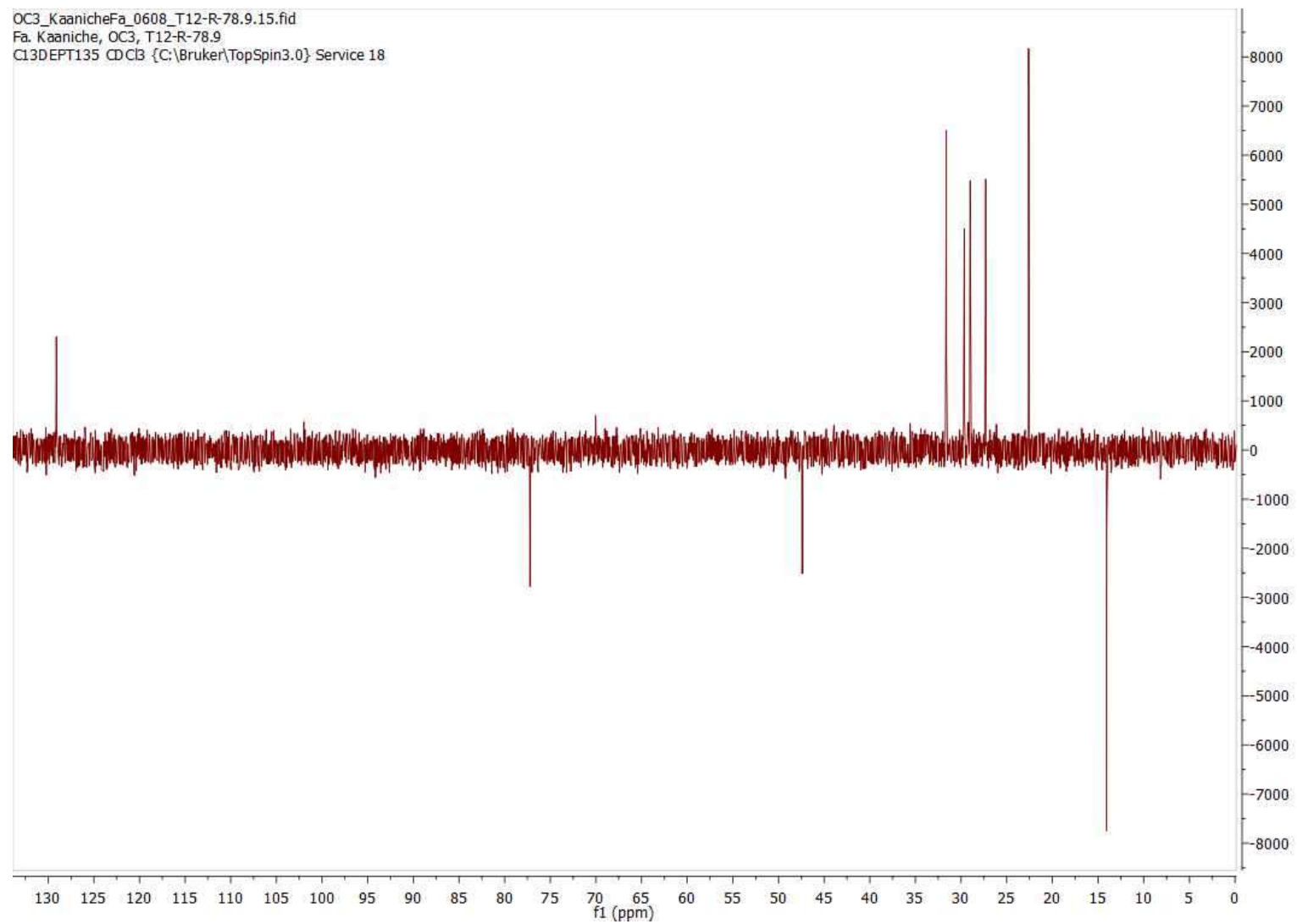

Supplement: S9 Fig — (PDF) [file pone.0217627.s011.pdf]

**S10 Fig.** H,H-COSY spectrum (CDCl<sub>3</sub>, 500 MHz) of Hexylitaconic acid (**2**)

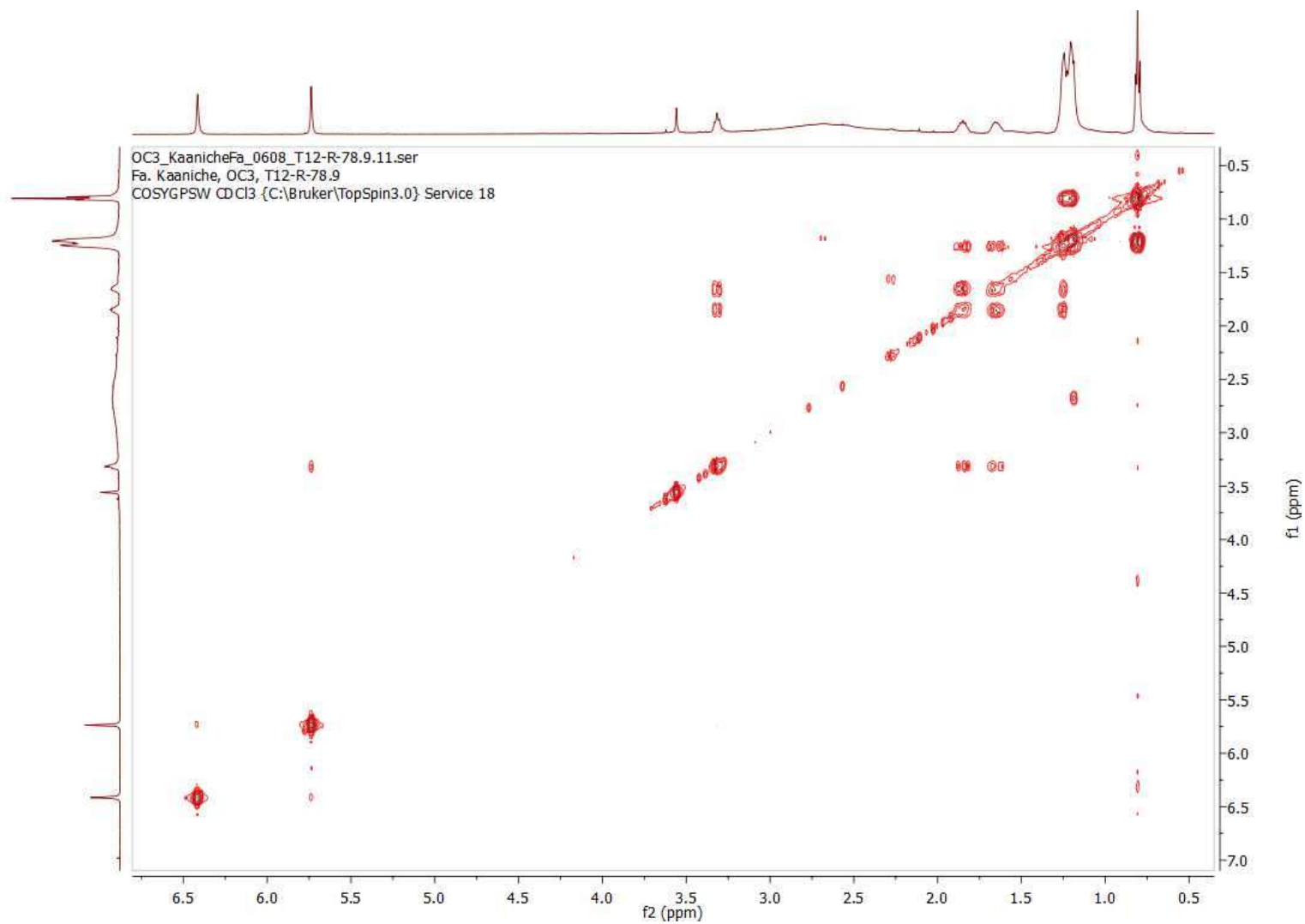

Supplement: S10 Fig — (PDF) [file pone.0217627.s012.pdf]

**S11 Fig.** HMQC spectrum (CDCl<sub>3</sub>, 500 MHz) of Hexylitaconic acid (**2**)

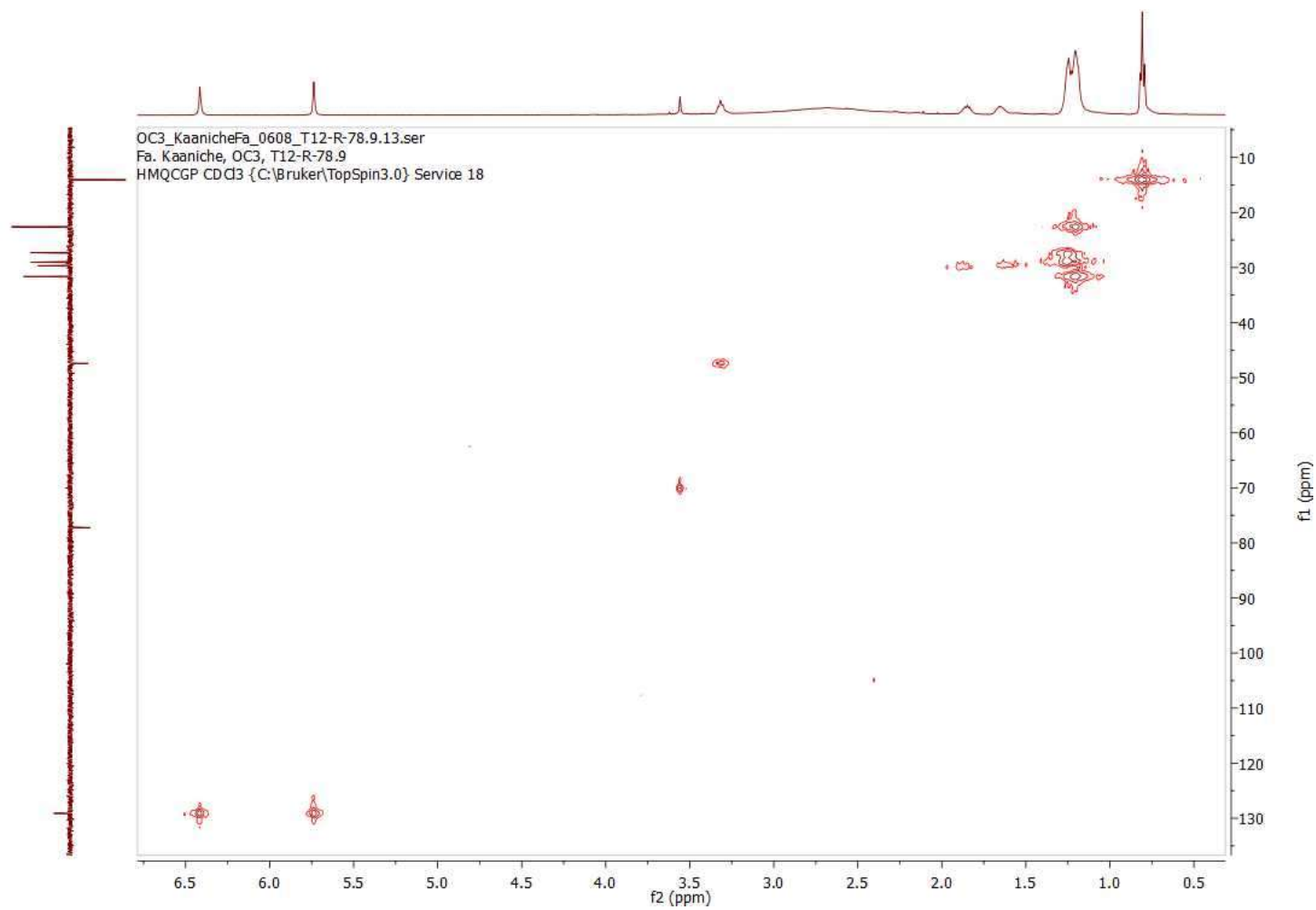

Supplement: S11 Fig — (PDF) [file pone.0217627.s013.pdf]

**S12 Fig.** HMBC spectrum (CDCl<sub>3</sub>, 500 MHz) of Hexylitaconic acid (**2**)

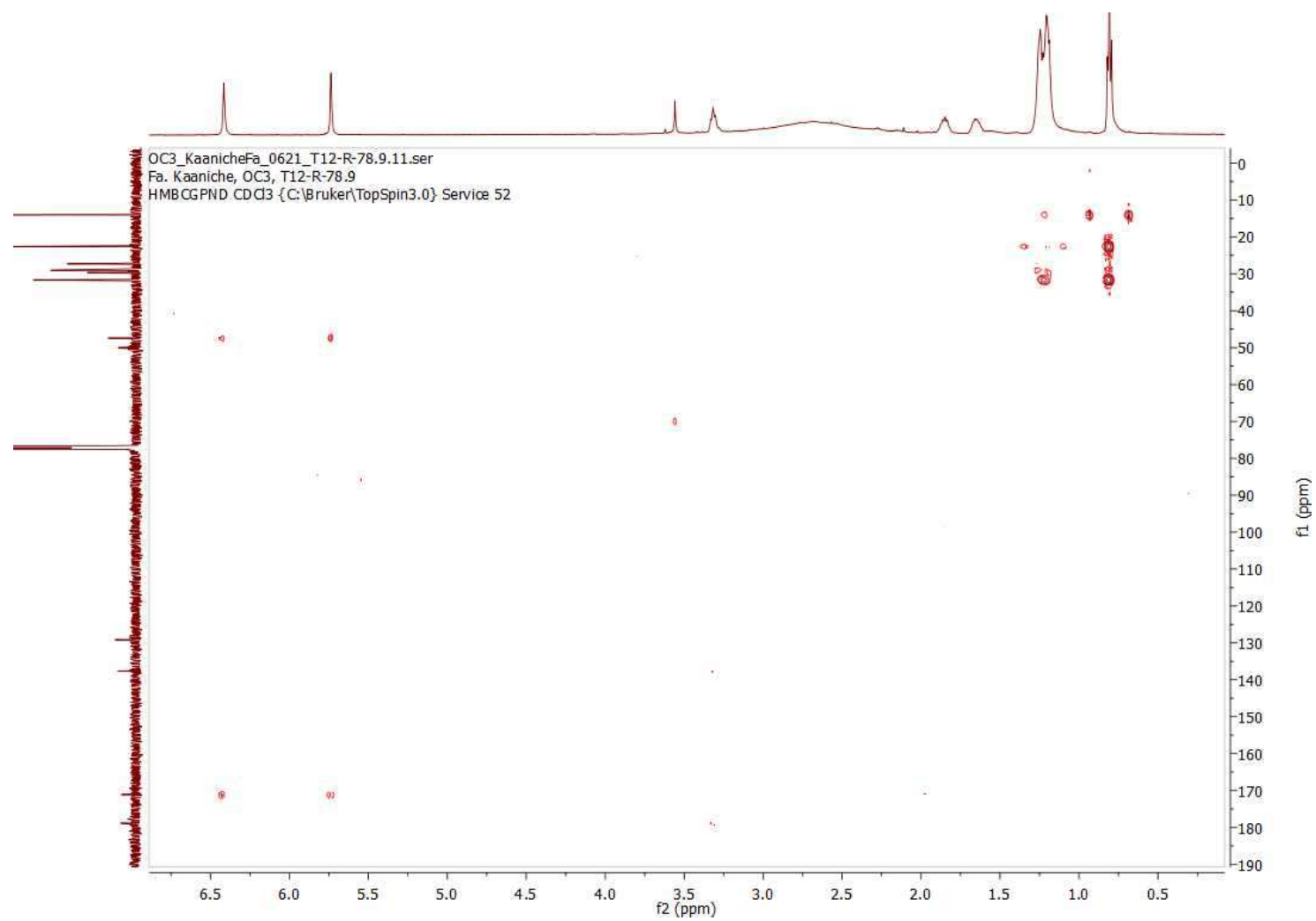

Supplement: S12 Fig — (PDF) [file pone.0217627.s014.pdf]

**S13 Fig.**  $^1\text{H}$  NMR spectrum ( $\text{CDCl}_3$ , 500 MHz) of ergosterol (**3**)

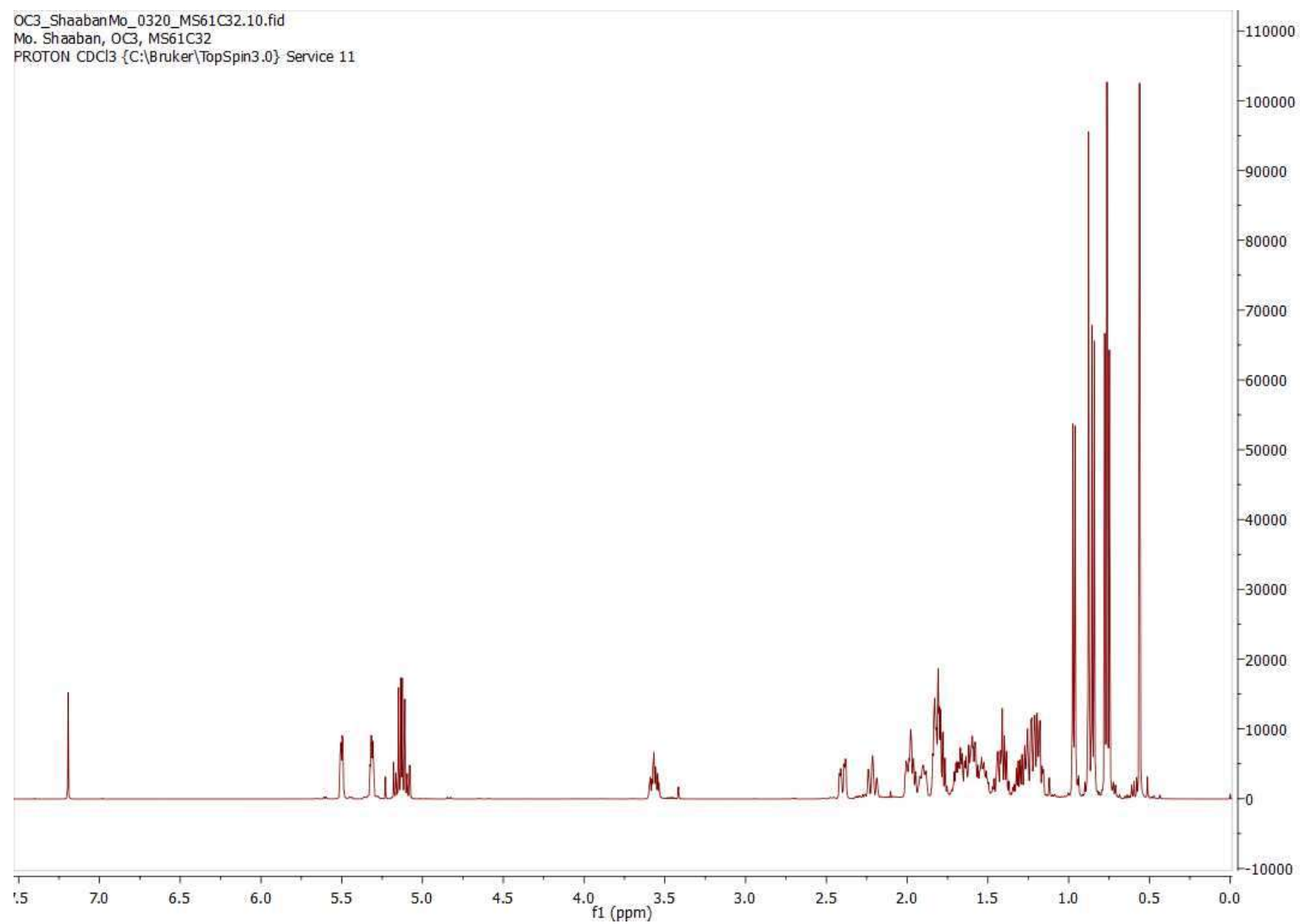

Supplement: S13 Fig — (PDF) [file pone.0217627.s015.pdf]

**S14 Fig.**  $^{13}\text{C}$  NMR spectrum ( $\text{CDCl}_3$ , 125MHz) of ergosterol (**3**)

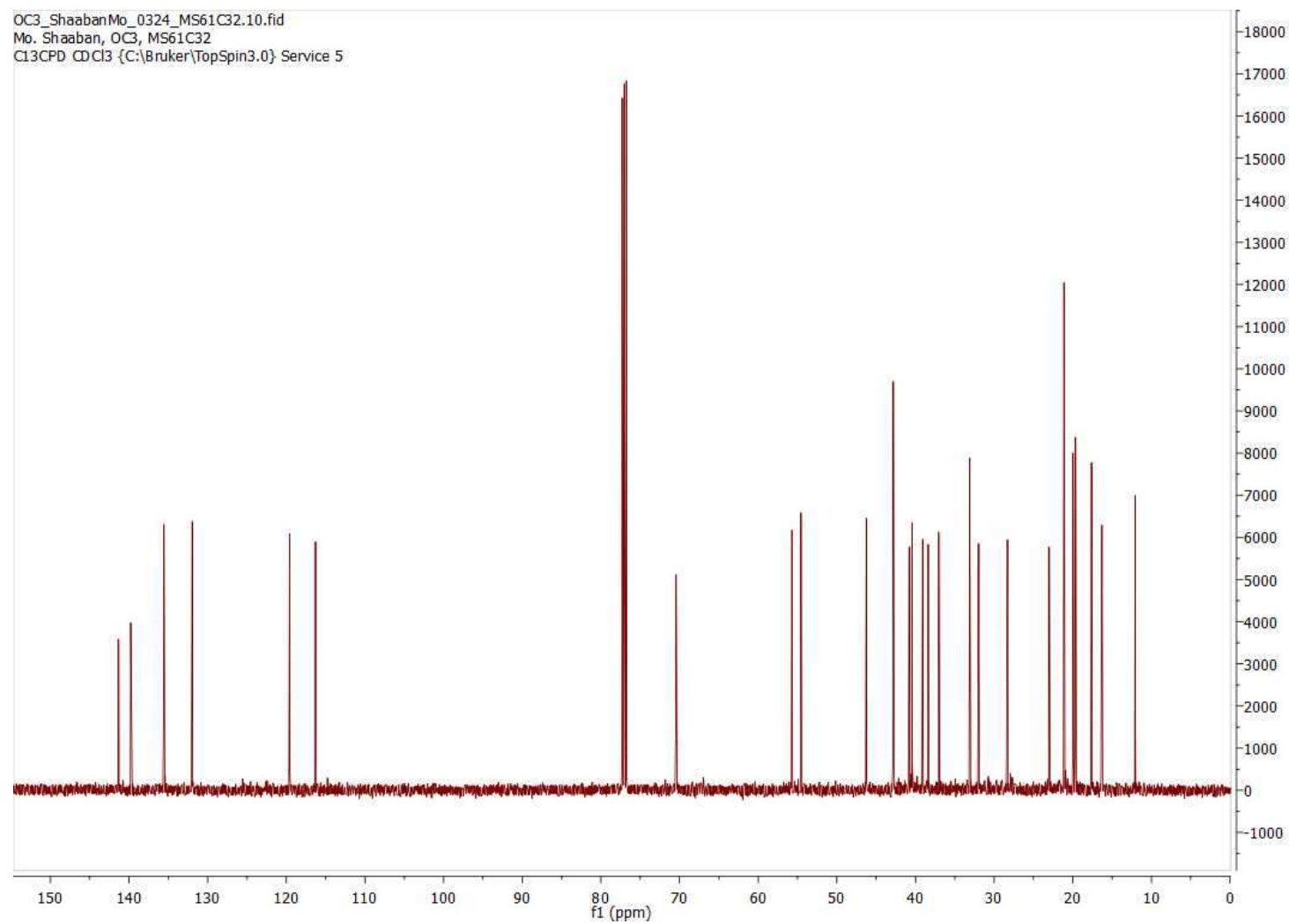

Supplement: S14 Fig — (PDF) [file pone.0217627.s016.pdf]
